# Optofluidic transport and assembly of nanoparticles using an all-dielectric quasi-BIC metasurface

**DOI:** 10.1038/s41377-023-01212-4

**Published:** 2023-07-28

**Authors:** Sen Yang, Justus C. Ndukaife

**Affiliations:** 1 Department of Electrical and Computer Engineering, Vanderbilt University, Nashville, TN USA; 2 Interdisciplinary Materials Science, Vanderbilt University, Nashville, TN USA; 3 Department of Mechanical Engineering, Vanderbilt University, Nashville, TN USA

**Keywords:** Nanophotonics and plasmonics, Optical manipulation and tweezers, Optofluidics

## Abstract

Manipulating fluids by light at the micro/nanoscale has been a long-sought-after goal for lab-on-a-chip applications. Plasmonic heating has been demonstrated to control microfluidic dynamics due to the enhanced and confined light absorption from the intrinsic losses of metals. Dielectrics, the counterpart of metals, has been used to avoid undesired thermal effects due to its negligible light absorption. Here, we report an innovative optofluidic system that leverages a quasi-BIC-driven all-dielectric metasurface to achieve subwavelength scale control of temperature and fluid motion. Our experiments show that suspended particles down to 200 nanometers can be rapidly aggregated to the center of the illuminated metasurface with a velocity of tens of micrometers per second, and up to millimeter-scale particle transport is demonstrated. The strong electromagnetic field enhancement of the quasi-BIC resonance increases the flow velocity up to three times compared with the off-resonant situation by tuning the wavelength within several nanometers range. We also experimentally investigate the dynamics of particle aggregation with respect to laser wavelength and power. A physical model is presented and simulated to elucidate the phenomena and surfactants are added to the nanoparticle colloid to validate the model. Our study demonstrates the application of the recently emerged all-dielectric thermonanophotonics in dealing with functional liquids and opens new frontiers in harnessing non-plasmonic nanophotonics to manipulate microfluidic dynamics. Moreover, the synergistic effects of optofluidics and high-Q all-dielectric nanostructures hold enormous potential in high-sensitivity biosensing applications.

## Abstract (web summary)

We propose an innovative optofluidic system, employing an all-dielectric quasi-BIC metasurface, that achieves subwavelength scale optical heating and fluid motion control via precisely tuning wavelengths within several nanometers range. Long-range transport and rapid assembly of nanoparticles are presented.

## Graphical abstract

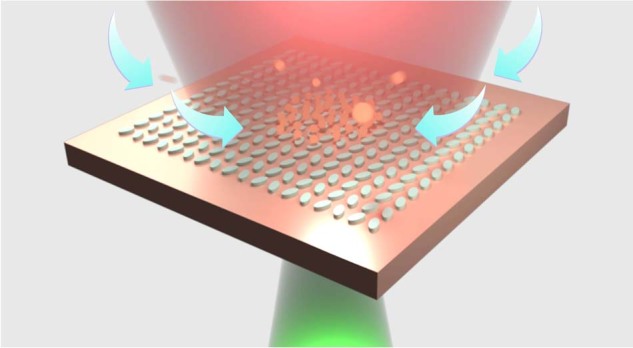

## Introduction

Controlling the long-range transport of fluids has been a fundamental requirement in microfluidic systems, from standard flow cell assays to lab-on-a-chip devices. Traditionally, pressure-driven control techniques and syringe pumps have been widely used in microfluidics^1^. The evident scale mismatch between the microfluidic system and the bulky control system has inspired significant efforts to develop integrated micrometer scale control techniques^2^. Among them, one promising approach to achieve integrated control of particle and fluid motion is to use light to control the flow of fluids, particularly at the micrometer scale, i.e., optofluidics^3^. The buoyancy-driven toroidal convection generated by heating the water with a laser beam can help transport and concentrate particles, but the flexibility to control the convection flow is limited^4,5^. One solution to achieve fluid manipulation is to use localized thermal gradients induced by light illumination^3^.

Metal nanostructures, when illuminated by light at their plasmonic resonance, can tightly confine the energy to subwavelength scales in the vicinity of the nanostructures. The enhanced light absorption from the intrinsic (Ohmic) losses of metals has been considered as side effects such as limited quality factor (Q)^6^, and reduced trapping stability in plasmonic nanotweezers due to undesired thermal heating effects^7^. Recently, however, scientists have realized that this enhanced light absorption can efficiently turn metal nanostructures into nanosources of heat, inspiring the study of thermoplasmonics^8–10^. This finding has found numerous applications in nanotechnology, namely, for photothermal cancer therapy^11^, targeted drug delivery^12^, solar-powered steam generation^13^, as well as nanoscale control of temperature distribution^8,14^ and thereby for optofluidics. Single^15,16^ and arrays^17,18^ of metal nanostructures have been studied to control the convection-driven dynamics. However, the surface of metal nanostructures may have an unnecessarily high temperature which can be harmful to particles touching the surface^19^.

All-dielectric nanostructures, on the other hand, have been rapidly developing for the past decade mainly because they are relieved of parasitic Ohmic losses inherent by plasmonic nanostructures^20^. Many works have leveraged its low light absorption and thereby negligible Joule heating to avoid undesired fluid motion and/or positive (repulsive) thermophoresis which can deteriorate the optical trapping stability in plasmonic nanotweezers^21^, such as by leveraging silicon dimers^22^ and anapole-assisted nanoantennas^23–25^. Very recently, however, an emerging new field of all-dielectric thermonanophotonics focuses on controlling subwavelength scale optical heating by precisely tuning optical losses in dielectrics^26^. In ref. ^26^, it was identified that temperature-gradient-driven microfluidic flows may also be controlled by dielectric nanoparticles. In this work, for the first time, we experimentally demonstrate the synergistic effects of optofluidic transport and particle aggregation using an all-dielectric metasurface enabled by quasi-bound states in the continuum (quasi-BIC) resonances.

**Fig. 1:**
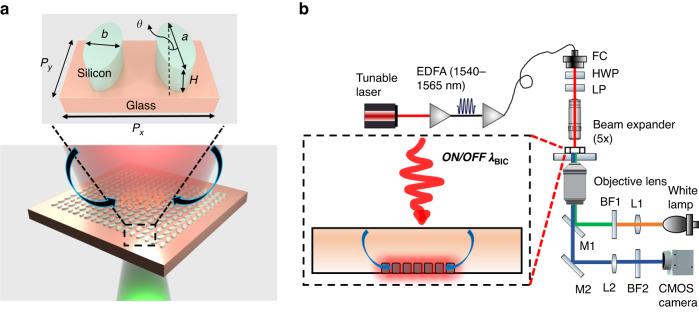
**Working principle and experimental facility. a** Schematic of the system. When the metasurface is off-resonance, the laser heating of the bulk water induces buoyancy-driven flow, transporting and aggregating particles to the center of the illuminated region. When the quasi-BIC is excited, additional heat sources come from the heat dissipation of the water layer close to the resonators. The thermal-induced flow velocity is increased up to three times. The flow is represented by the two arrows above the nanoantennas. Inset: a unit cell of the metasurface. The geometrical parameters: periods, ${P}_{x}=950\,{\rm{nm}}$, ${P}_{y}=778\,{\rm{nm}}$; $a=532\,{\rm{nm}}$, $b=192\,{\rm{nm}}$, $H=190\,{\rm{nm}}$, $\theta =10^\circ$. **b** Experimental set-up used for excitation of the quasi-BIC metasurface and imaging of the motion of suspended tracer particles. L1 and L2, focusing lenses; M1 and M2, mirrors; BF1 and BF2, bandpass filters used to filter light used for excitation of the fluorescent particles and light transmitted for imaging on the camera, respectively. Filtered fluorescent illumination is passed through the objective lens (10× or 40×) and focused on the sample. EDFA, Erbium-doped fiber amplifier used to amplify the power of the input laser; FC fiber collimator, HWP half wave-plate used to rotate the polarization direction of the laser beam, LP linear polarizer. The metasurfaces and fluorescent tracer particles are visualized on a complementary metal-oxide-semiconductor (CMOS) camera by collecting signals through the same objective lens

**Fig. 2:**
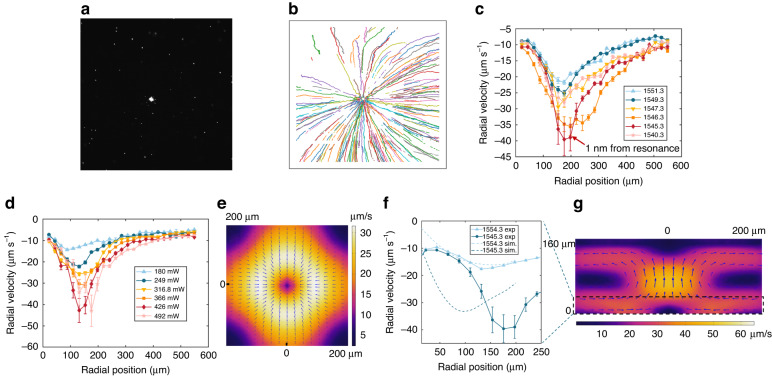
**Experiment and simulation results for particle transport**. All data are obtained under a 10× objective lens. The field of view is ~900 μm. **a** Representative particle aggregation when a collimated laser beam is illuminated on the metasurface. **b** Representative particle trajectory map extracted from sequential 600 frames of a recorded video. The frame rate is 10 frames per second. The empty region at the center indicates that particle movement is trivial in this area, corresponding to the aggregated particle cluster. The result shows that the flow is directed radially inwards towards the center of the laser spot and serves as a powerful means to concentrate suspended particles to the vicinity of the metasurface. It’s noted that some particle trajectories are interruptted at the edge of the metasurface region. This is due to the low transmittance of silicon in the visible range, which dims the fluorescence of these tracer particles, making them hard to be tracked. **c** Scaling of experimentally measured radial flow velocity with laser wavelength. The negative sign represents the inward direction. The error bar shows the standard error of the mean. The laser power fluctuates around 420 mW. The position where velocity reaches a maximum is slightly farther away from the center for wavelengths closer to the resonance. We attribute this to the stronger positive (repulsive) thermophoresis in the lateral direction due to the stronger heating effect. **d** Scaling of experimentally measured radial flow velocity with laser power. The same repulsive phenomenon is observed for higher laser power. **e** Simulated flow velocity distributions in the near-resonant condition of the *xy*-plane (5 μm above the substrate) and **g** of the *xz*-plane. Color map shows the velocity magnitude and superimposed arrows show the direction of the flow vectors. Radial velocities in the black dash box region (**g**) are averaged to obtain the dash lines in (**f**). **f** Simulated (dash lines) and measured (solid lines) radial flow velocity for near-resonance (1545.3 nm) and off-resonance (1554.3 nm). The measured maximum velocities for 1545.3 nm and 1554.3 nm are about 45 μm s^-1^ and 17 μm s^-1^, respectively

**Fig. 3:**
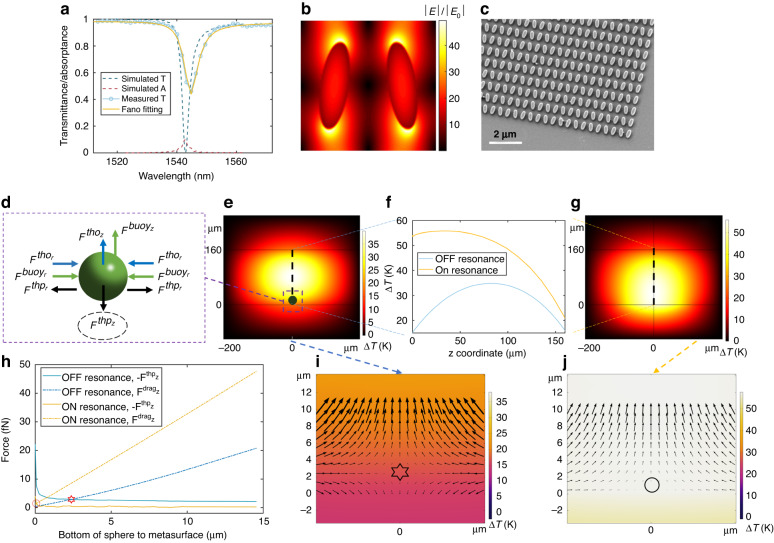
**Optical characterization and thermal simulation**. **a** Simulated and measured spectra of the metasurface. The resonance positions are 1548.9 nm and 1544.8 nm for the simulation and measurement, respectively. The sample shows a larger transmittance (46.1%) and a larger linewidth (6.3 nm) compared to the simulations (0% and 3.0 nm, respectively) attributed to the finite size and fabrication imperfections. The well-overlapped Fano fitting curve (yellow solid line) with the measured transmittance spectrum validates a typical quasi-BIC resonance. **b** Electric field enhancement distribution within one unit cell. The maximum electric field enhancement factor is 49.4, i.e., 2440 times for the intensity enhancement, supporting strong enhancement of water absorption. **c** Representative scanning electron microscopy image of the fabricated metasurface. The array size is 500 μm. **d** Depiction of the major forces acting on the trapped particles suspended in deionized water for the off-resonant condition. tho, thermo-osmosis; buoy, buoyancy-driven convection; thp, thermophoresis. **e** Simulated temperature field distribution of the *xz* plane for off-resonant conditions and **g** for on-resonant conditions. Black dash lines mark the paths from which the spatial temperature rise curves in (**f**) are extracted. **f** Temperature rise in *z* direction for off-resonant and on-resonant conditions. *z* = 0 is the surface of the glass substrate. **h** Forces exerted on a 500 nm PS bead. ${F}^{{{\rm{drag}}}_{{\rm{z}}}}$ corresponds to the Stokes drag force resulting from the fluid flow, which is composed of ${F}^{{{\rm{buoy}}}_{{\rm{z}}}}$ and ${F}^{{{\rm{tho}}}_{{\rm{z}}}}$. The direction of the thermophoretic force is reversed as $-{F}^{{{\rm{thp}}}_{{\rm{z}}}}$ for a better comparison. A correction factor is used for the thermophoresis due to the hydrodynamic boundary effects^58,59^. The star and circle symbols represent the balance point for off and on-resonant conditions, respectively. **i** Zoom-in temperature field distribution of the *xz* plane, superimposed with vectors of the total force exerted on a 500 nm PS bead for off-resonant conditions and **j** on-resonant conditions. The star symbol in (**i**) and the circle symbol in (**j**) denote the same symbols in (**h**)

The concept of BIC, first proposed in quantum mechanics by von Neumann and Eugene Wigner in 1929^27^ has rapidly emerged as a powerful approach for realizing high Q and strong field enhancement in dielectric metasurfaces^28^. The dielectric quasi-BIC metasurfaces are highly promising as they provide high-Q resonances comparable to photonic crystals^29^, and strong field enhancements comparable to or even higher than those reported in plasmonic nanostructures. A variety of applications have been demonstrated in this field including lasing^30,31^, biosensing^32–36^, and nonlinear harmonic generation^37^. Among these designs, the intrinsic losses of materials have been identified as a critical issue for the quasi-BIC modes^38,39^. We reported that the loss of the surrounding environment (refractive index of water at 1.55 μm, *n* = 1.31 + 0.00013i) can strongly affect the quasi-BIC resonance^40^. The absorptance from water in the proximity of the metasurface composed of elliptical silicon resonators can be as high as 45%, even though the loss from the resonators made by silicon is negligible. As shown by our recent work in which the water absorption in a single photonic crystal cavity served as a heat source for electrothermal effects^41^, in this work, we instead discuss how to leverage the heat dissipation due to water absorption in a quasi-BIC system to engineer the microfluidic flow (see Fig. 1). Approaching the resonance, the total heat generation comprises of the global absorption by the bulk water in the microfluidic chamber as well as the heat dissipation from the water layer close to the resonators which serves as the local heat sources. Due to the high-Q property of quasi-BIC resonances, this localized heating effect makes the system capable of achieving precise control of the temperature field distribution by simply tuning the wavelength within several nanometers range, as discussed in Fig. 3. Moreover, the flow velocity can be increased up to about three times in comparison to that of the off-resonant condition. Therefore, the flow velocity can also be precisely controlled by slightly tuning the wavelength from off to on resonant conditions, as discussed in Fig. 2. Such precise control of the temperature field and flow dynamics by tuning wavelengths is not achievable in traditional plasmonic systems due to their low-Q properties. Suspended particles can be rapidly transported (with velocities of 10 to 10^2^ micrometer-per-second) from distances of up to millimeter-scale and aggregated at the center of the laser spot by the flow. The transported particles are then confined close to the metasurface by positive thermophoresis. Fluorophore-labeled tracer polystyrene (PS) beads with a size down to 200 nm are examined and we expect the same effects can be achieved for particles below 100 nm. Finally, we showcase different particle aggregation distributions when adding a cationic surfactant (cetyltrimethylammonium chloride) to the nanoparticle colloid by tuning the metasurface to off or on resonances. This verifies the temperature field distribution is modified by the quasi-BIC resonance. As such, our work opens a new field for controlling thermal-induced microfluidic dynamics at the subwavelength scale by non-plasmonic nanostructures.

## Results

### Working principle and experimental set-up

Our system comprises an all-dielectric quasi-BIC metasurface sitting on the substrate of a microfluidic chamber and illuminated with a collimated and linearly polarized laser beam, as shown in Fig. 1b. The laser spot follows Gaussian distribution, and the diameter is around 300 μm after it is shrunken through a beam expander by five times. The laser power is amplified by an Erbium-doped fiber amplifier (EDFA) such that an output of hundreds of milliwatts can be obtained. Relatively strong water absorption falls within the working range of the EDFA. The metasurface is composed of elliptical silicon nanoantennas arranged in a zigzag array on a glass substrate (see Fig. 1a). When the input wavelength is away from the quasi-BIC resonance (${\lambda }_{{\rm{BIC}}}$), the laser beam is transmitted after passing through the 160 μm thick water film (i.e., chamber height). The laser heating induces buoyancy-driven natural convection, which transports particles to the center of the illuminated substrate. Particles are confined close to the substrate in the axial direction (i.e., normal to the substrate) by the positive thermophoresis^42^, as depicted in Fig. 1a. When the input wavelength is approaching ${\lambda }_{{\rm{BIC}}}$, the quasi-BIC resonance is excited. Highly localized field enhancement (see Fig. 3b) in the tip-to-tip gaps induces strong light absorption in the adjoining water medium. These hotspots then serve as local heat sources inducing strong temperature gradients for manipulating microfluidic dynamics at the subwavelength scale. Besides the overall increased temperature which induces a faster buoyancy-driven flow, these local hot spots also induce strong thermo-osmotic flow^43,44^ due to the large lateral temperature gradient. Therefore, particles can be aggregated much more rapidly.

### Particle aggregation

The representative aggregation of particles when illuminating the metasurface is shown in Fig. 2a. We used 500 nm fluorophore-labeled PS beads as tracer particles to visualize the microfluidic flow. Experimental demonstrations using 200 nm PS beads are shown in V3 of the supplementary videos. The experimental videos are processed using an open-source particle tracking analysis package called *Trackpy*^45^. Representative particle trajectories are presented in Fig. 2b. It can be found that particles are rapidly transported towards the laser spot center and aggregated close to the substrate. The empty region at the center of the trajectory map represents a stagnation zone, i.e., the aggregated particle cluster. Since the aggregated particle cluster only takes ~0.13% of the area of the whole metasurface and the refractive index contrast between PS and water is low, we note that the impact of the particles to the quasi-BIC resonance was neglected. The experimentally measured, angularly averaged radial velocities obtained from the particle tracking analysis are shown in Fig. 2c and d. As shown in Fig. 2c and V1 of the supplementary videos, the fluid radial velocity when the collimated laser beam is illuminated on the metasurface increases rapidly as the input wavelength approaches the resonance (1544.3 nm). The results show a maximum flow radial velocity of 45 μm s^-1^ at 1545.3 nm, about two times larger than that at 1551.3 nm. This indicates that the flow velocity can be precisely controlled over a wide range by simply tuning the wavelength within a ±7 nm bandwith due to the high-Q attribute of the quasi-BIC resonance. This is not achievable in plasmonic arrays. For example, in ref. ^17^, the wavelength distance for a 2-times difference of the maximum velocity is almost 100 nm, which is two orders of magnitude larger compared to our case. Therefore, the transport and aggregation of particles can be manipulated quite flexibly and benefit from the local thermal gradients caused by the quasi-BIC metasurface.

As shown in Fig. 2c, d, the radial flow velocity close to the metasurface initially increases from a radial distance of more than ~ 550 μm (limited by the field of view) until it reaches its maximum at a radial distance of ~180 μm from the particle cluster. Inward from this position, the radial velocity decreases towards the center of the laser spot. Thus, we expect particles within at least a range of 550 micrometer-scale radius can be rapidly captured and transported to the center of the illuminated region. The scaling of the flow velocity with laser power is shown in Fig. 2d. The flow velocity rises when the input power is increased. The particle tracking analysis is not applicable when the input power is larger than 500 mW as the particles move too fast to be accurately located.

To understand the physics of the observed thermal-induced microfluidic dynamics in this system, we numerically solved the flow field by a commercially available finite element method software package (COMSOL Multiphysics). We consider that two mechanisms contribute to the thermal-induced flows in this system: the buoyancy-driven convection and thermo-osmotic flow^43,44^. The velocity field distribution is determined from the solution of the incompressible Navier–Stokes equation given by

1 $${\rho }_{0}({\bf{u}}({\bf{r}})\cdot \nabla ){\bf{u}}({\bf{r}})+\nabla p({\bf{r}})-\eta {\nabla }^{2}{\bf{u}}({\bf{r}})={\bf{F}}$$

where $\nabla \cdot {\bf{u}}=0$, ${\rho }_{0}$, *p*(**r**) and *η* are fluid density, pressure, and dynamic viscosity, respectively; and **F** is the force per unit volume acting on the fluid element. For the buoyancy-driven convection, we employ the Boussinesq approximation given by^15,17^

2 $${{\boldsymbol{F}}}_{{\rm{buoy}}}=g{\rho }_{0}\beta (T)\left[T({\boldsymbol{r}})-{T}_{0}\right]$$

where *g* and *β*(*T*) are the gravitational constant, and thermal expansion coefficient of water, respectively. For the thermo-osmotic flow, we apply a slip velocity to the thin fluid layer close to the surface of the substrate given by^43,44^

3 $${v}_{\parallel }=\chi \frac{{\nabla }_{\parallel }T}{T}$$

where $\chi$ is the thermo-osmotic coefficient dependent on the properties of the liquid-solid interface (*ζ* potential, Hamaker constant, etc.) and ${\nabla }_{\parallel }T$ is the temperature gradient parallel to the surface. The related temperature field distributions and simulations will be discussed in the next section (see Fig. 3).

The simulated velocity distributions in the *xy*-plane near the substrate and in the *xz*-plane are shown in Fig. 2e, g, respectively. The flow close to the substrate brings particles inwards towards the center of the illuminated area and drags particles away from the surface in the axial direction (*z* direction). Due to the large depth of field of the 10× objective lens, the measured radial velocities are averaged from particles that appear within 30 μm from the surface (see S5 of SI). Simulation results implementing the similar averaging are compared with experimental results in Fig. 2f. The measured maximum flow velocity at 1545.3 nm reaches around three times of that at 1554.3 nm (10 nm away from resonance). For the off-resonant condition, the simulation agrees well with the measurement. For the near-resonant condition, the trend of the simulation remains consistent with the measurement despite the underestimation. We attribute this difference mainly to the underestimation of the thermo-osmosis contribution. Our simulations show that the thermo-osmotic flow is weak in the off-resonant condition as the lateral temperature gradient is low for the large Gaussian-distributed laser spot and it is insensitive to the changes of the thermo-osmotic coefficient $\chi$. However, when the quasi-BIC mode is excited, the thermo-osmotic flow becomes an important contributor to the total transport flow velocity and significantly increases it. This is due to the strong ${\nabla }_{\parallel }T$ provided by the hotspots near the nanoantennas. As shown in Fig. 3b, the electric field distribution in each tip-to-tip gap should induce a stronger ${\nabla }_{\parallel }T$ and consequently a stronger thermo-osmotic flow. However, in our simulations, we use a pillar with uniform heat distribution to represent such a non-trivial heat distribution for computational simplicity (see S1 of SI for details). This assumption likely underestimates the local temperature gradients and thus leads to a lower flow velocity. Furthemore, our estimation for the zeta potential, *ζ*, of the water-substrate interface may also play a part.

### Particle dynamics

Up to now, we have demonstrsted the boosted particle transport and aggregation assisted by the quasi-BIC metasurface. To elucidate the particle dynamics, the following section focuses on the dynamics of particles^46,47^ when on and off resonances. We start from the optical characterization of the metasurface. The simulated and measured spectra of the metasurface are shown in Fig. 3a, respectively. For an infinite array, no light is transmitted and the absorption of water (red dash line in Fig. 3a) accounts for the 10% drop in the reflected power. In our experiments, each metasurface is a 500 μm by 500 μm square array, large enough compared with the laser spot diameter. The finite array size and the fabrication uncertainties (see Fig. 3c for the scanning electron microscopy image) suppress the performance of the resonance mode and increase the background noise during measurement. Therefore, a higher transmittance (from 0% to 46.1%) and a reduced Q (from 500 to 250) are observed in the measured spectra. We expect this reduced Q impairs the heating effect while our experiments show that it is still strong enough to modify the whole temperature field distribution. The large near-field electric field enhancement confined to the tip-to-tip gaps of the nanoantennas contributes to the strong heating effects, as shown in Fig. 3b. We highlight that plasmonic nanostructures generally possess a smaller mode volume and tighter field confinement compared to their all-dielectric counterparts. However, this does not necessarily translate to superior control of optofluidic motion based on buoyancy-driven convection^15^. A stronger flow requires an array of plasmonic nanostructures to harness the collective heating effect with a spatially distributed thermal landscape^14,17^. On the other hand, our quasi-BIC metasurface provides spatially distributed heating combined with high-Q resonances to achieve precise control of the temperature distribution (see Fig. 3e–g) and optofluidic flow (see Fig. 2c) by varying the incident wavelength. It is also worth noting that the BIC mode we employ is a symmetry-protected mode and is sensitive to the incident angle and the array size^30,48^. This is the key reason we choose to use a collimated laser beam instead of a focused laser beam by an objective lens.

To understand the modulation of the heat distribution by the quasi-BIC resonance, we numerically solved the temperature field by COMSOL Multiphysics. The temperature field in the system is determined by solving the steady-state heat equation given by

4 $$\nabla \cdot \left[-\kappa \nabla T({\bf{r}})+\rho {c}_{{\rm{p}}}T({\bf{r}}){\bf{u}}({\bf{r}})\right]=q({\bf{r}})$$

The first term on the left corresponds to heat conduction, while the second term corresponds to heat convection depending on the velocity of the flow. *T*(**r**) and **u**(**r**) are the spatial temperature and fluid velocity field, respectively; *κ*, *ρ* and *c*_*p*_ are the thermal conductivity, density, and heat capacity of the materials, respectively; and *q*(**r**) is the heat source density given by the heat dissipated per unit volume. In our system, *q*(**r**) is comprised of two parts. The first part comes from the light absorbed by the bulk water modeled by^14^

5 $$q\left({\boldsymbol{r}}\right)={P}_{0}\frac{{\alpha }_{{\rm{c}}}}{2\pi {\sigma }^{2}}{{\rm{e}}}^{-\left(\frac{{r}^{2}}{2{\sigma }^{2}}\right)}{{\rm{e}}}^{-{\alpha }_{{\rm{c}}}{\rm{z}}}$$

6 $${\alpha }_{{\rm{c}}}=\frac{4\pi \bar{\kappa }}{\lambda }$$

where *P*_0_ is incident laser power, *λ* is laser wavelength, ${\alpha }_{c}$ is the attenuation coefficient of water, $\bar{\kappa }$ is the imaginary component of the refractive index of water. σ is defined by ${w}_{0}=2\sigma$ which is the waist radius of the Gaussian beam. The second part comes from the light absorbed by the water near the nanoantennas when the quasi-BIC resonance is excited. For computational simplicity, we approximate the heat dissipation obtained from electromagnetic calculations as uniform at each hotspot while taking into account the spatial Gaussian distribution of the incident laser beam (see S1 of SI for details).

To better compare the heat distributions when on and off resonances, we simulate the temperature fields for the two cases, as shown in Fig. 3e, g, respectively. The temperature drops from the middle of the chamber down to the substrate. Therefore, particles experience a positive thermophoretic force^42^ which pushes particles towards the substrate and acts as the main mechanism to confine particles in the axial direction. Since the thermophoretic force^49^ is proportional to ${S}_{T}\nabla T$ where ${S}_{T}$ is the Soret coefficient, the confinement stability depends on the temperature gradient in the axial direction. Although the highest temperature rise increases from 38 K to 56 K, the location of the highest temperature rise changes from the chamber center in the off-resonant condition to the vicinity of the substrate under the on-resonant condition. This is due to the strong heating effects near the resonators, as depicted in Fig. 3f. Therefore, the temperature gradient in the axial direction is decreased in the on-resonant condition, leading to decrease of the downward thermophoretic force.

**Fig. 4:**
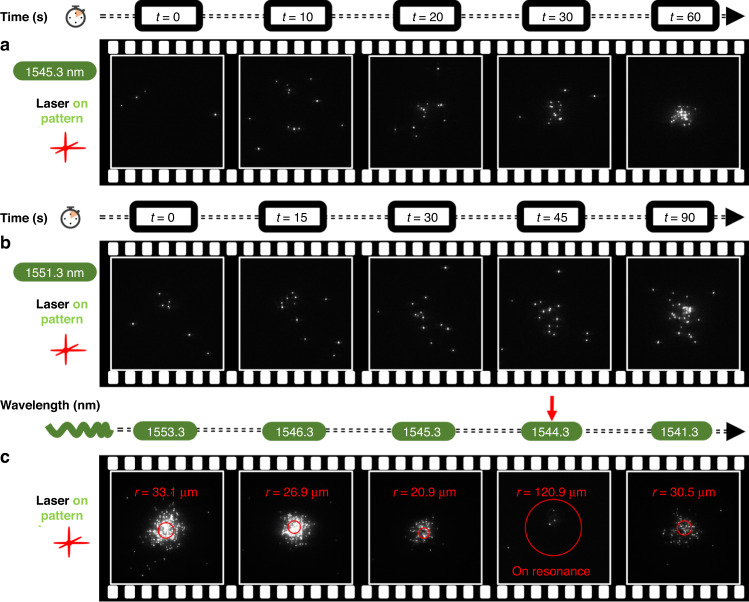
**Experiment results for particle aggregation**. All data are obtained under a 40× objective lens. The laser power fluctuates around 270 mW. **a** Evolution of particle aggregation with illumination time when the input wavelength is near-resonance (1545.3 nm) and **b** off-resonance (1551.3 nm). Particles are aggregated more rapidly and packed more tightly in the near-resonant condition. **c** Evolution of particle aggregation with illumination wavelength. The cluster is packed more tightly when approaching resonance, while particles start to lost when very close to resonance. No particles can be aggregated on resonance. Particles are aggregated again when away from resonance

Based on the aforementioned temperature field analysis, we describe the particle dynamics as follows. The key forces acting on the trapped particles suspended in deionized water are depicted in Fig. 3d. The positive thermophoretic force ${F}^{{\rm{thp}}}$ pushes particles from the middle down towards the substrate in the axial direction. The drag force ${F}^{{\rm{buoy}}}$ from the buoyancy-driven convection brings particles towards the center in the lateral direction and takes particles away from the substrate in the axial direction. The thermo-osmotic flow is generated due to the lateral temperature gradient close to surface. The related drag force ${F}^{{\rm{tho}}}$ moves particles in the same directions as the buoyancy-driven convection. In the lateral direction, particles can be confined at the center by these centripetal flows^50^. In the axial direction, these two upward drag forces balance with the downward positive thermophoresis at around 2.4 μm above the substrate, as marked by the star symbol in Fig. 3h, i. When a particle is transported to within 2.4 μm from the substrate, it can be further pushed down and confined close to the substrate as the total force points down. However, under the on-resonant condition, besides the increased buoyancy-driven convection, the thermo-osmotic flow becomes much stronger due to the increased lateral temperature gradient while the axial thermophoretic force ${F}^{{{\rm{thp}}}_{{\rm{z}}}}$ becomes weaker attributed to the decreased axial temperature gradient. Such a situation leads to a shift of the balance position to only around 70 nm above the substrate, as shown by the circle symbol in Fig. 3h, j. This observation indicates that it’s hard to confine a particle unless the particle is very close to the substrate, as the total force points up above 70 nm. Therefore, we expect a lower stability for particle aggregation when on resonance. In addition, when the quasi-BIC mode is excited, the short-range optical gradient force derived from the highly enhanced electric field may be another mechanism to pull particles to the hotspots. However, we note that in this work, the thermal forces and increased Brownian motion dominate the dynamics of the particles due to the strong heating effects. Thus, when the laser wavelength was tuned to ${\lambda }_{{\rm{BIC}}}$, the particle aggregation was observed to disappear as depicted in Fig. 4c. More detailed discussions can be found in S7 of SI.

Next, we experimentally investigate the dynamics of particles in our system. We compare the evolution of particle aggregation with illumination time when the input wavelength is near-resonant (1545.3 nm) and off-resonant (1551.3 nm), as shown in Fig. 4a, b. It can be found that particles are concentrated in a faster manner for near-resonant illumination. More specifically, the time to concentrate particles to reach a similar number of aggregated particles in the near-resonant condition is only two-thirds of that for the off-resonant condition. This agrees with the aforementioned flow velocity measurements. Moreover, the particle cluster is packed more tightly in the near-resonant condition as shown in the last frame of Fig. 4a, b. We attribute this to the stronger thermal-induced flow directed towards the center due to stronger heating effects, which exerts larger drag forces on the particles. Notably, approaching the resonance condition does not necessarily improve particle aggregation. The particle cluster at different input wavelengths is shown in Fig. 4c. The cluster is packed more tightly from 1553.3 nm to 1546.3 nm as approaching the resonance, represented by the effective particle distribution region (see S2 of SI) marked by the red circles. However, the concentrated particles start to be lost when the input wavelength is closer to resonance (1545.3 nm) in which the number of particles is decreased, while the cluster is even more tightly packed. For the on-resonant condition (1544.3 nm), the particles can not be aggregated anymore. We note that the slightly different resonance position from the spectra measurement (Fig. 3a) comes from the sample loading process as this symmetry-protected BIC mode is sensitive to the incident angle^34^. Three nanometers away from resonance (1541.3 nm), the particle cluster appears again. This observation clearly proves our aforementioned analysis of the particle dynamics. The mechanism for these phenomena is two-fold. First, at resonance (or very close to resonance), the temperature gradient in the axial direction near the metasurface is no longer strong enough to stably confine particles to the substrate by the positive thermophoresis, as seen in Fig. 3e–g. Second, the thermal-induced fluid flow from both the buoyancy-driven convection and thermo-osmosis is stronger, resulting in stronger drag forces in the axial direction to take particles away from the substrate, as seen in Fig. 3h–j. We anticipate this can be improved by suppressing the quasi-BIC mode properly to reduce the heating effects from the high electric field enhancement, for example, a larger tilt angle *θ* in our case.

Finally, to further validate our analysis, we added a cationic surfactant, cetyltrimethylammonium chloride (CTAC) to the nanoparticle colloid to indirectly investigate the temperature field distribution when on and off resonances. CTAC has been widely used for reversing the sign of the effective Soret coefficient, i.e., attracting particles towards hot regions^51^. CTAC molecules adsorbed on the PS particle surface can form a positively charged molecular double layer. Simultaneously, CTAC molecules self-assemble into micelles when above the critical micelle concentration (0.13–0.16 mM). As reported in ref. ^51^, when the CTAC concentration is below ~2 mM, a thermoelectric field is generated to attract particles towards the hot region. When the CTAC concentration is above ~2 mM, the depletion of the CTAC micelles plays a key role in attracting particles due to the generation of the depletion-attraction force (DAF). In brief, DAF is an osmotic pressure exerted on particles to attract them towards hot regions due to the concentration gradient of micelles generated from the migration of micelles from hot to cold regions^52^. In our case, we consider the latter mechanism as the CTAC concentration in our experiments was 5 mM.

**Fig. 5:**
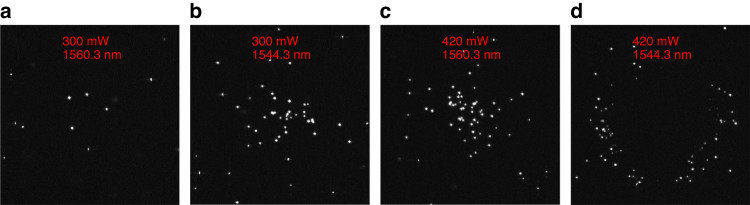
**Experiment results for CTAC solution**. CTAC concentration is 5 mM. All data are obtained under a 10× objective lens. **a** Particles are hard to accumulate at low power (300 mW) and **c** aggregated at high power (420 mW) for the off-resonant condition (1560.3 nm). **b** Particles are aggregated at low power (300 mW) and **d** localized as a ring at high power (420 mW) for the on-resonant condition (1544.3 nm). The aggregation is not as tight as that of previous experiments in deionized water mainly due to the increased thermophoresis repelling particles from the center

Figure 5 shows different particle distributions for on and off-resonance cases, respectively. This verifies our aforementioned discussions that the temperature field distribution is modified by the quasi-BIC resonance of the nanoantennas. For the off-resonant condition (see S6 of SI for the force depiction), particles are hard to be aggregated on the substrate at low laser power (Fig. 5a) as they are attracted towards the hot center at the middle of the chamber (see Fig. 3e) by the DAF. At high power (Fig. 5c), however, particles are aggregated again as the positive thermophoresis overcomes the DAF to push the particles down. On the other hand, for the on-resonant condition, since the hot center is close to the substrate, particles are aggregated at low power (Fig. 5b) but show a ring-like localization at high power (Fig. 5d and V2 of the supplementary videos). This transition from accumulation to ring-like distribution is due to the interplay between the thermophoresis and DAF and has been reported by several research groups where the hot spot was located at the top wall of the chamber and the concentration of the added polymers was varied^52,53^. In these works, the chamber height was usually less than 10 μm to suppress thermal convections. In a very recent work, Simon et al. reported that the transition can also happen between low and high laser power^54^. We emphasize that the positive thermophoretic force in this experiment was strong enough to repel particles in the lateral direction to generate the ring-like distribution when the quasi-BIC mode is excited and the temperature is higher. This enhanced positive thermophoresis is attributed to the following reason. The magnitude and even the sign of the *ζ* potential of particles can be significantly modified by the adsorbed surfactant molecules^55^. In our experiments, the *ζ* potential of the 500 nm PS beads is modified from -40 mV suspended in deionized water to +88 mV with the CTAC concentration of 5 mM (see S3 of SI). If we neglect the permittivity and salinity gradients, the thermophoretic mobility^44,55^ of the particle is proportional to *ζ*^2^. In this case, the natural thermophoretic force repelling particles from the hot center increases up to around five times. We expect this ring-like distribution can be beneficial for concentrating particles at cool regions as well as separating different particles^53^.

## Discussions

In summary, we have introduced and demonstrated the subwavelength scale control of the temperature field and fluid motion in an all-dielectric system. Owning to the high quality factor and strong electromagnetic field enhancement of the quasi-BIC mode, we present precise control of the fluid velocity up to three times by simply tuning laser wavelength within several nanometers. We also show long-range (millimeter-scale) and rapid (tens of micrometers per second) particle transport and aggregation. The undesired reduction of aggregation stability on resonance is observed and attributed to the modified temperature field altered by the strong heating effects from resonant quasi-BIC nanoantennas. This can be improved by slightly suppressing the quasi-BIC mode, for example by illuminating with the wavelength slightly away from the resonance or increasing the tilt angle of the elliptical nanoantennas. By implementing a physical model, we numerically show how the quasi-BIC resonance altered the temperature field and fluid dynamics at the subwavelength scale. Moreover, after adding a cationic surfactant, CTAC, to the nanoparticle colloid, the totally different particle aggregation distributions for on and off-resonant conditions further validate our analysis. The ring-like particle aggregation that arises on resonance also provides potential applications for assembling and separating particles in low-temperature regions.

In addition, we propose that this system can become a powerful tool in colloid science and life science. Although we show aggregation of polystyrene beads down to 200 nm, we expect the same effect can be applied for smaller particles such as those in the sub-100 nm size regime. This can offer many opportunities in biology and medicine fields such as for concentration and detection of extracellular vesicles and viruses. Finally, the combination of optofluidics and high-Q all-dielectric nanostructures can have great potential in boosting the sensitivity of biosensors, benefitting from synergistic effects of effective particle aggregation and strong electromagnetic field enhancement in the near field. For example, several works have reported promising sensing performances employing all-dielectric quasi-BIC metasurfaces as label-free nanophotonic biosensors^32,34,35^. However, in these systems, the analytes are immobilized on the sensor surface through either dropping droplets, spin coating, or pressure-driven flow. We expect that the ability of our system to concentrate particles suspended in liquid can further push down the detection limit of such quasi-BIC-based biosensors.

## Materials and methods

### Metasurface fabrication

All samples were fabricated on square glass wafers (Präzisions Glas & Optik GmbH, with a thickness of 0.7 mm) with a side length of 15 mm. Amorphous silicon (a-Si) films with a thickness of 190 nm (measured by visible-NIR ellipsometry) were deposited onto the glass wafers by plasma-enhanced chemical vapor deposition (Trion Orion), followed by the spin coating of a double layer of polymethyl methacrylate (PMMA) resist of different molecular weights (495 K A2 and 950 K A4) and resistive physical vapor deposition (Angstrom Amod) of a 10 nm thick chromium conduction layer. The 500 μm by 500 μm patterns were transferred to the resist by 30 keV electron beam lithography (Raith eLiNE), after which the sample was dipped first in chromium etchant (Transene Chrome Mask Etchant 9030) to remove the conduction layer and then in the MIBK/IPA 1:3 (Kayaku Advanced Materials) for development. A chromium hard mask (10 nm thickness) was vertically deposited via electron-beam physical vapor deposition (Angstrom Amod) followed by wet-chemical lift-off in NMP 1165 remover (Microposit) on a 80°C hot plate overnight. Finally, patterns were subsequently transferred onto the underlying a-Si layer by fluorine-based dry plasma etching (Oxford PlasmaPro 100 Cobra) with the chromium hard mask residue removed by the chromium etchant.

### Surface treatment

To prevent the fluorophore-labeled polystyrene beads (Sigma-Aldrich, negatively charged in deionized water) from getting too close to the sample surface due to electrostatic interactions and then stuck by the Vander Waals force in the deionized water, we treated the sample surface to make it negatively charged. The sample was first dipped in 3 mM polystyrene sulfonate (PSS, Sigma-Aldrich) polymer colloid for 10 min followed by a rinse with deionized water. Then the sample was dipped in 1 M potassium chloride (KCL) solution for 10 min followed by a rinse with deionized water.

### Chip encapsulation

The treated sample was carefully covered by a thin glass coverslip separated by a double-layer adhesive film (see S4 of SI) with an average thickness of 160 μm that sealed the microfluidic chamber. Polystyrene bead colloids with different concentrations were injected into the chamber by syringes.

### Multiphysics simulations

The temperature and flow field distributions were calculated by the commercially available software COMSOL Multiphysics 5.6.

The dimension of the whole simulation region was set as 200 μm × 200 μm × 360 μm. To mimic the encapsulated chip, the materials from top to bottom were glass, water, and glass, with thicknesses of 100 μm, 160 μm, and 100 μm, respectively. A four-fold symmetry (i.e., only a quarter of the whole region was simulated, as shown in Fig. S1 of SI) was applied to reduce the memory requirement and save the simulation time. In other words, the whole area we calculated for was 400 μm × 400 μm. Two stationary steps solved *Heat Transfer in Solids and Fluids* and *Laminar Flow* studies sequentially.

The *Heat Transfer in Solids and Fluids* study solved the temperature field distribution. The *Temperature* (293.15 K) boundary condition was set for all the outer boundaries. The thermal conductivity of water and glass was set as 0.598 and 1.38 W m^−1^ K^−1^, respectively. The heat capacity of water and glass was set as 4200 and 840 J kg^−1^ K^−1^, respectively. As we have discussed in the main text, the heat sources are composed of two components: absorption from the water bulk (global) and the water layer close to the resonators (local). Therefore, we defined the two types of heat sources separately. For details of how the two heat sources are set, see section S1 of SI for details. The Soret coefficient of a 500 nm PS bead^56^ was set as 1.0 K^−1^.

The *Laminar Flow* study solved both the buoyancy-driven flow and thermo-osmotic flow induced by the temperature gradient and only the water region was included. The upper boundary was set as *Wall* with no slip while the surrounding boundaries were set as *Open Boundary*. As discussed by Martin Fränzl et al. ^44^, to account for the thermo-osmotic slip flow the lower boundary was defined as a wall with a slip velocity. The *Thermal slip coefficient* ${\sigma }_{T}$ was set as 0.02 assuming a surface *ζ* potential of −100 mV^57^. The expression of *Volume Force* was expressed below which represents the buoyancy-driven convection force:

7 $${F}_{z}=g{\rho }_{0}\beta (T)\left[T({\bf{r}})-{T}_{0}\right]\hat{z}$$

where *T*_0_ = 293.15 K; *g*, *β*(*T*) are the gravitational constant, and thermal expansion coefficient of water, respectively.

## Supplementary information

SI

V1

V2

V3

## References

[1] Iverson BD, Garimella SV (2008). Recent advances in microscale pumping technologies: a review and evaluation. Microfluidics Nanofluidics.

[2] Squires TM, Quake SR (2005). Microfluidics: fluid physics at the nanoliter scale. Rev. Mod. Phys..

[3] Chen J (2020). Thermal optofluidics: principles and applications. Adv. Opt. Mater..

[4] Liu C (2019). Low-cost thermophoretic profiling of extracellular-vesicle surface proteins for the early detection and classification of cancers. Nat. Biomed. Eng..

[5] Flores-Flores E (2015). Trapping and manipulation of microparticles using laser-induced convection currents and photophoresis. Biomed. Opt. Express.

[6] Khurgin JB, Boltasseva A (2012). Reflecting upon the losses in plasmonics and metamaterials. MRS Bull..

[7] Zhang YQ (2021). Plasmonic tweezers: for nanoscale optical trapping and beyond. Light Sci. Appl..

[8] Baffou G, Quidant R (2013). Thermo-plasmonics: using metallic nanostructures as nano-sources of heat. Laser Photonics Rev..

[9] Baffou G, Cichos F, Quidant R (2020). Applications and challenges of thermoplasmonics. Nat. Mater..

[10] Hong CC (2021). Electrothermoplasmonic trapping and dynamic manipulation of single colloidal nanodiamond. Nano Lett..

[11] Rastinehad AR (2019). Gold nanoshell-localized photothermal ablation of prostate tumors in a clinical pilot device study. Proc. Natl Acad. Sci. USA.

[12] Timko BP, Dvir T, Kohane DS (2010). Remotely triggerable drug delivery systems. Adv. Mater..

[13] Neumann O (2013). Solar vapor generation enabled by nanoparticles. ACS Nano.

[14] Baffou G (2013). Photoinduced heating of nanoparticle arrays. ACS Nano.

[15] Donner JS (2011). Plasmon-assisted optofluidics. ACS Nano.

[16] Hong C, Yang S, Ndukaife JC (2019). Optofluidic control using plasmonic TiN bowtie nanoantenna [invited]. Opt. Mater. Express.

[17] Roxworthy BJ (2014). Understanding and controlling plasmon-induced convection. Nat. Commun..

[18] Ciraulo B (2021). Long-range optofluidic control with plasmon heating. Nat. Commun..

[19] Li M, Lohmüller T, Feldmann J (2015). Optical injection of gold nanoparticles into living cells. Nano Lett..

[20] Kuznetsov AI (2016). Optically resonant dielectric nanostructures. Science.

[21] Crozier KB (2019). Quo vadis, plasmonic optical tweezers?. Light Sci. Appl..

[22] Xu Z, Song WZ, Crozier KB (2018). Optical trapping of nanoparticles using all-silicon nanoantennas. ACS Photonics.

[23] Conteduca D (2021). Exploring the limit of multiplexed near-field optical trapping. ACS Photonics.

[24] Hernández-Sarria JJ, Oliveira ON, Mejía-Salazar JR (2021). Toward lossless infrared optical trapping of small nanoparticles using nonradiative anapole modes. Phys. Rev. Lett..

[25] Hong, I. et al. Anapole-assisted near-field optical trapping. Proceedings of SPIE PC12198, Optical Trapping and Optical Micromanipulation XIX. San Diego, CA, USA: SPIE, 2022, PC121981E.

[26] Zograf GP (2021). All-dielectric thermonanophotonics. Adv. Opt. Photonics.

[27] von Neumann J, Wigner EP (1929). Über merkwürdige diskrete Eigenwerte. Physikalische Z..

[28] Joseph S (2021). Bound states in the continuum in resonant nanostructures: an overview of engineered materials for tailored applications. Nanophotonics.

[29] Chen ZH (2022). Observation of miniaturized bound states in the continuum with ultra-high quality factors. Sci. Bull..

[30] Kodigala A (2017). Lasing action from photonic bound states in continuum. Nature.

[31] Huang C (2020). Ultrafast control of vortex microlasers. Science.

[32] Tittl A (2018). Imaging-based molecular barcoding with pixelated dielectric metasurfaces. Science.

[33] Yesilkoy F (2019). Ultrasensitive hyperspectral imaging and biodetection enabled by dielectric metasurfaces. Nat. Photonics.

[34] Leitis A (2019). Angle-multiplexed all-dielectric metasurfaces for broadband molecular fingerprint retrieval. Sci. Adv..

[35] Jahani Y (2021). Imaging-based spectrometer-less optofluidic biosensors based on dielectric metasurfaces for detecting extracellular vesicles. Nat. Commun..

[36] Yang S (2022). Engineering electromagnetic field distribution and resonance quality factor using slotted Quasi-BIC metasurfaces. Nano Lett..

[37] Liu ZJ (2019). High-*Q* quasibound states in the continuum for nonlinear metasurfaces. Phys. Rev. Lett..

[38] Yoon JW, Song SH, Magnusson R (2015). Critical field enhancement of asymptotic optical bound states in the continuum. Sci. Rep..

[39] Bochek DV (2022). Bound states in the continuum versus material losses: Ge_2_Sb_2_Te_5_ as an example. Phys. Rev. B.

[40] Yang S (2021). Nanoparticle trapping in a quasi-BIC system. ACS Photonics.

[41] Yang S (2023). Multiplexed long-range electrohydrodynamic transport and nano-optical trapping with cascaded bowtie photonic crystal nanobeams. Phys. Rev. Lett..

[42] Braun D, Libchaber A (2002). Trapping of DNA by thermophoretic depletion and convection. Phys. Rev. Lett..

[43] Bregulla AP (2016). Thermo-osmotic flow in thin films. Phys. Rev. Lett..

[44] Fränzl M, Cichos F (2022). Hydrodynamic manipulation of nano-objects by optically induced thermo-osmotic flows. Nat. Commun..

[45] Crocker JC, Grier DG (1996). Methods of digital video microscopy for colloidal studies. J. Colloid Interface Sci..

[46] Shi YZ (2018). Sculpting nanoparticle dynamics for single-bacteria-level screening and direct binding-efficiency measurement. Nat. Commun..

[47] Shi YZ (2018). Nanometer-precision linear sorting with synchronized optofluidic dual barriers. Sci. Adv..

[48] Jin J (2019). Topologically enabled ultrahigh-Q guided resonances robust to out-of-plane scattering. Nature.

[49] Piazza R, Parola A (2008). Thermophoresis in colloidal suspensions. J. Phys.: Condens. Matter.

[50] Stoev ID (2021). Highly sensitive force measurements in an optically generated, harmonic hydrodynamic trap. eLight.

[51] Lin LH (2018). Opto-thermoelectric nanotweezers. Nat. Photonics.

[52] Jiang HR (2009). Manipulation of colloids by a nonequilibrium depletion force in a temperature gradient. Phys. Rev. Lett..

[53] Maeda YT, Buguin A, Libchaber A (2011). Thermal separation: interplay between the soret effect and entropic force gradient. Phys. Rev. Lett..

[54] Simon, D., Thalheim, T. & Cichos, F. Optical manipulation of single DNA molecules by depletion interactions. Proceedings of SPIE PC12198, Optical Trapping and Optical Micromanipulation XIX. San Diego, CA, USA: SPIE, 2022, PC121981C.

[55] Lin LH (2017). Interfacial-entropy-driven thermophoretic tweezers. Lab a Chip.

[56] Braibanti M, Vigolo D, Piazza R (2008). Does thermophoretic mobility depend on particle size?. Phys. Rev. Lett..

[57] Bastos, D. & de las Nieves, F. J. On the zeta-potential of sulfonated polystyrene model colloids. Trends in Colloid and Interface Science VII. Heidelberg, Germany: Steinkopff, 37–44 (1993).

[58] Lin LH (2017). Thermophoretic tweezers for low-power and versatile manipulation of biological cells. ACS Nano.

[59] Würger A (2016). Hydrodynamic boundary effects on thermophoresis of confined colloids. Phys. Rev. Lett..

