# Supplementary material for: Optofluidic transport and assembly of nanoparticles using an all-dielectric quasi-BIC metasurface
Source: Light Sci Appl. 2023 Jul 28;12:188. doi: 10.1038/s41377-023-01212-4 (PMC10382587; doi:10.1038/s41377-023-01212-4)
Supplement: Supplementary file 1 — SI [file 41377_2023_1212_MOESM1_ESM.pdf]

Supporting Information

Optofluidic transport and assembly of nanoparticles using an all-dielectric quasi-BIC metasurface

Sen Yang[3,2,1], Justus C. Ndukaife[3,4,2,1*]

[1]Vanderbilt Institute of Nanoscale Science and Engineering, Vanderbilt University, Nashville, TN, USA 37235
[2]Interdisciplinary Materials Science, Vanderbilt University, Nashville, TN, USA 37235
[3]Department of Electrical and Computer Engineering, Vanderbilt University, Nashville, TN, USA 37235
[4]Department of Mechanical Engineering, Vanderbilt University, Nashville, TN, USA 37235

Authors contact details:

Sen Yang: sen.yang@vanderbilt.edu

[*]Justus C. Ndukaife: justus.ndukaife@vanderbilt.edu, +1 (615)875-1662

Pages: S1 – S7

Figures: S1 – S6

Video descriptions: V1 – V3

Sections: S1 – S8

S1: Additional details on heat source settings and optical simulations

S1.1 Heat source settings

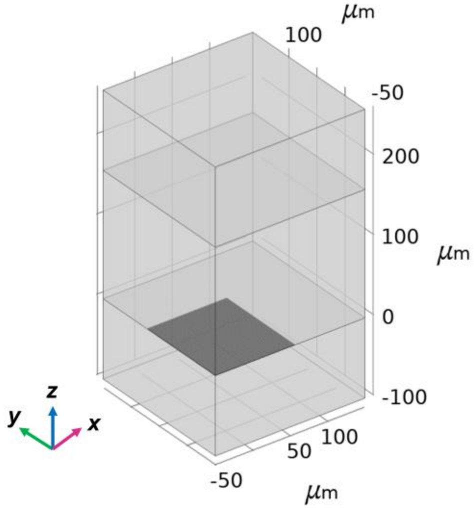

Fig. S1. COMSOL model for Multiphysics simulations. By adding the *Symmetry* boundary condition on the inner *yz* and *xz* planes (planes facing readers), only a quarter of the whole area is shown and simulated.

For the global heat source, the heat source type was chosen as *General source*. Water absorption from the bulk follows the Beer–Lambert law. Therefore, the heat source density (W/m^3) when light passing through the water bulk was modeled using the expression in Eq. (S1) while taking into account the paraxial approximation formula[1] of the Gaussian distribution of the incident laser beam[2]

$$q(\boldsymbol{r}) = P_0 \frac{\alpha_c}{2\pi\sigma^2} \mathrm{e}^{-\left(\frac{r^2}{2\sigma^2}\right)} \mathrm{e}^{-\alpha_c(H-z)}, \alpha_c = \frac{4\pi\bar{\kappa}}{\lambda} \qquad \text{(S1)}$$

where H = 160 μm is the chamber height; P_0 = 420 mW is the incident laser power; the attenuation coefficient in the water bulk medium is $\alpha_c = \frac{4\pi\bar{\kappa}}{\lambda}$, $\bar{\kappa}$ is the imaginary component of the refractive index of water ($\bar{\kappa} = 1e^{-4}$); and the waist radius of the Gaussian beam is defined as $w_0 = 2\sigma$ where w_0 was set as 150 μm estimated from the imaged laser spot. It's noted that the input light will be reflected back and pass through the water bulk again when the quasi-BIC mode is excited. To count in this, for the reflected light, P_0 was modified as P_0 × (1 - absorptance of water bulk) × (1 - absorptance of BIC – transmittance of BIC). Here, the absorptance of BIC at different wavelengths was obtained from FDTD simulations, and the absorptance of water bulk was calculated as 0.122 from the Beer–Lambert law. The transmittance of BIC was set as 0.80. It's noted that such measured average transmittance is higher compared with that in Fig. 3a of the main text. This is due to the degenerated performances of the samples after continuous experiments. The expression of the decay term in the *z* direction was also modified as $\mathrm{e}^{-\alpha_c z}$.

For the local heat sources, due to the memory limit, we assumed that the heat dissipation from the water layer close to the resonators in each tip-to-tip gap was uniform and we used periodically arranged cylinders (diameter = 160 nm, height = 250 nm) to represent these hot regions. Limited

by memory, the area of the cylinder array we simulated was about 55 μm × 55 μm, i.e., 220 μm × 220 μm considering the symmetry boundary condition (see Fig. S1). The heat source type was chosen as *Heat rate* and the heat power *P* was expressed as $P_0 \times 0.25 \times$ (1 – absorptance of water bulk) × absorptance of BIC $\times\, e^{-\left(\frac{r^2}{2\sigma^2}\right)}$ in which we added the Gaussian distribution term. More importantly, we further modified this expression to count in the normalization for such a discrete Gaussian distribution by multiplying by a coefficient of 2.16.

S1.2 Optical simulations

The numerical simulations of the spectra and field distributions were performed by commercially available software (Ansys Lumerical FDTD 2021 R2) using a finite-difference time-domain (FDTD) solver. *Periodic* boundary conditions were used in the *x* and *y* directions, while *PML* boundary conditions were used in the *z* direction. The *Anti-Symmetric* boundary conditions were applied in the *x* direction to save memory and simulation time. A *maximum mesh step* of 10 nm was set for the resonator region while a *mesh accuracy* of “5” was set for the rest. The *simulation time* was set to 10^5 fs and the *auto shutoff min* was set to 10^{-6} to ensure a good convergence of the calculations. The material properties were taken from the *Palik* dataset included in the software.

S2: Effective particle distribution region

The effective particle distribution region of an aggregated particle cluster was marked by a red circle as shown in Fig. 4c of the main text. The radius of such a circle for one frame was defined by calculating the average distance between pixels with their brightness not lower than 90% of the brightest pixel and the cluster center. The final radius shown in the figure was averaged over 1600 frames.

S3: Measurement of zeta potential

The zeta potential of the 500 nm polystyrene bead colloids was measured by *Zetasizer Ultra* (Malvern Panalytical Ltd). Fig. S2 shows a value of -40 mV for polystyrene beads suspended in deionized water and +88 mV for the CTAC (cetyltrimethylammonium chloride) concentration of 5 mM.

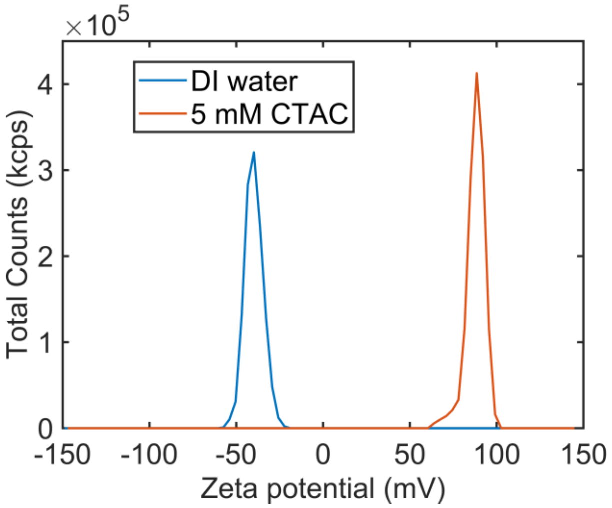

Fig. S2. Measured zeta potentials for polystyrene beads suspended in deionized water and in CTAC aqueous solution with a concentration of 5 mM.

S4: Thickness of the double-layer adhesive film

As shown in Fig. S3, the average thickness of the double-layer adhesive film used for sealing the chamber is about 160 μm. Therefore, we set the chamber height as 160 μm in the Multiphysics simulations.

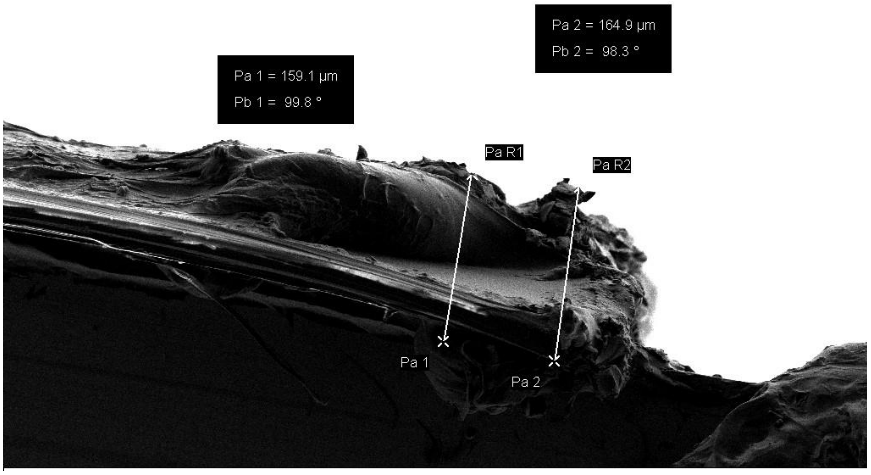

Fig. S3. Representative SEM image of a double-layer adhesive film showing the longitudinal cross-section.

S5: Depth of field of the 10x objective lens

As we have discussed in the main text, the measured radial velocities were estimated to be averaged from particles that appeared within about 30 μm from the metasurface. This was determined by the depth of field of the 10x objective lens used in the experiments. We investigated this by imaging particles immobilized on a glass wafer. The wafer with patterned particles was

gradually moved away from the focal plane through the piezo stage of the fluorescence microscope (Nikon Ti2). During this process, for a randomly chosen particle, its image became less clear, and the number of pixels it took increased. The *target particle size* was set to be 21 pixels (diameter) when processing the videos by the *trackpy* python package[3,4], which is roughly the 30 μm case shown in Fig. S4.

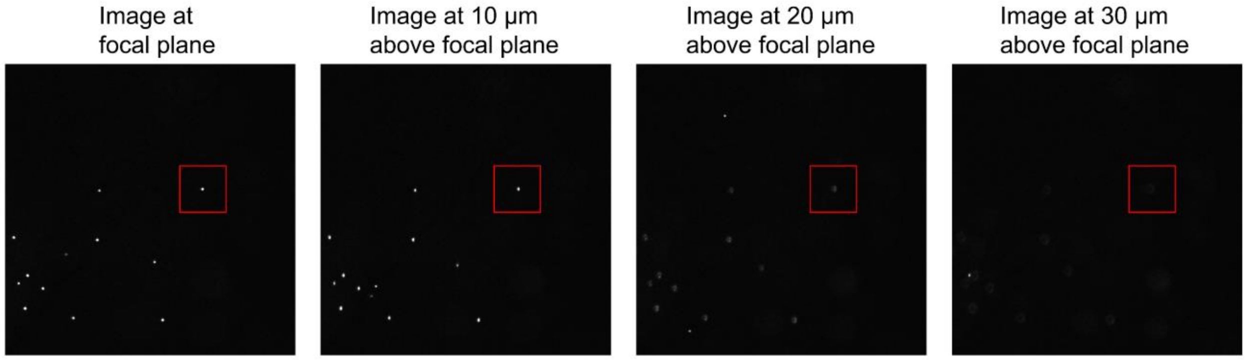

Fig. S4. Investigation of the depth of field. The same area was imaged every 1 μm when moving away from the focal plane. The studied particle enclosed in the red box in each image was randomly chosen at the beghining.

S6: Forces when adding CTAC

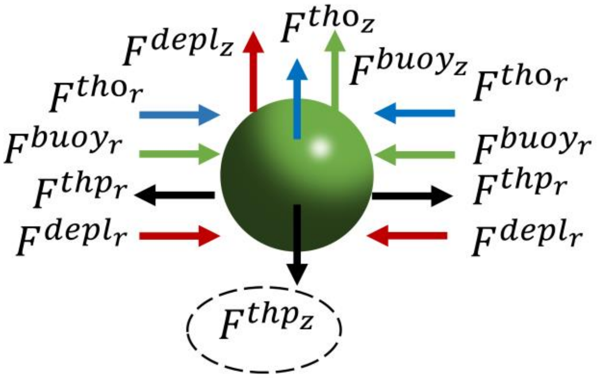

Fig. S5. Depiction of the major forces acting on the trapped particles suspended in the CTAC solution for the off-resonant condition. tho, thermo-osmosis; buoy, buoyancy-driven convection; thp, thermophoresis; depl, depletion-attraction force. As shown in Fig. 5a and 5c of the main text, at lower laser power, $F^{\mathrm{depl_z}} > F^{\mathrm{thp_z}}$ while at high laser power, $F^{\mathrm{depl_z}} < F^{\mathrm{thp_z}}$.

S7: Domination of thermal effects

Due to the absorption of water in this wavelength range, the chamber height is crucial in our system. Fig. S6a shows that both the temperature rise and the velocity of the convection flow drop rapidly as the chamber height decreases for off-resonant conditions. This is because less heat dissipation is generated from water absorption when light passes through a thinner chamber. Moreover, the convection flow is strongly suppressed in a thin chamber[6]. Since the chamber height in our experiments was 160 μm, such strong heating made thermal effects dominate the dynamics of particles, as discussed in Fig. 3 of the main text.

To consider the optical trapping of nanoparticles by such a quasi-BIC metasurface at ~ 1550 nm, as reported by our previous work[5], we need a low-power and low-heating condition[7]. As shown in Fig. S6b, with a chamber height of 1 μm and a laser power of 50 mW, the maximum temperature rise and flow velocity is suppressed to 1.7 K and 1.1 μm s^{-1} even under on-resonant conditions. In this situation, thermal effects such as the thermophoresis and Brownian motion are weak. Our simulations show that the optical gradient force that pulls a 500 nm PS bead 100 nm (bottom-to-surface distance) above the metasurface towards the hotspots is around 12 fN. While the maximum magnitude of the drag force and the thermophoretic force in the chamber is around 0.05 and 0.55 fN, respectively, much lower than the optical forces. In this case, it’s easy for a particle to overcome any thermally repelling forces and get close to a hotspot, where it can be optically trapped.

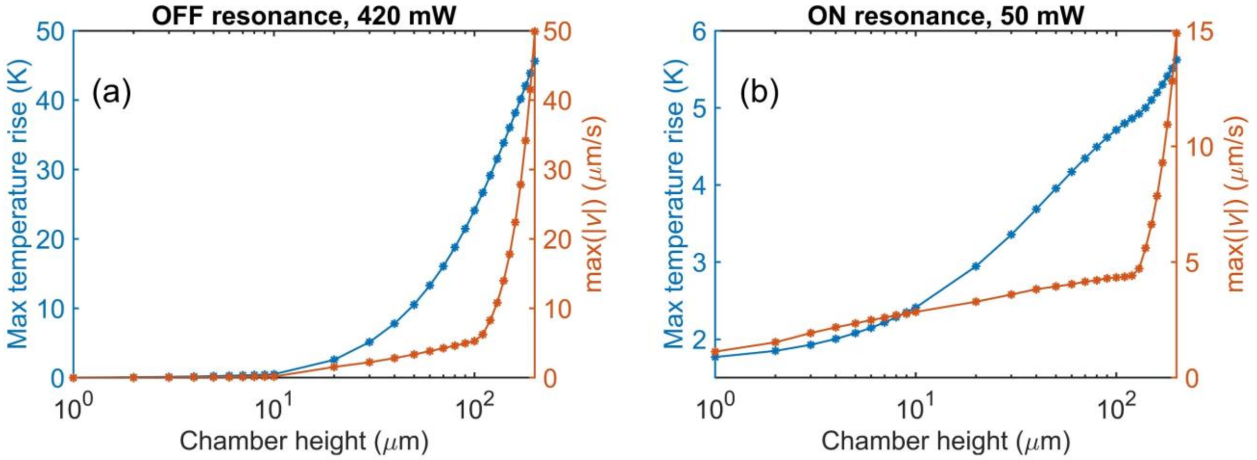

Fig. S6 (a) Simulated maximum temperature rise and magnitude of flow velocity with respect to the chamber height for off-resonant conditions with 420 mW laser power. (b) Simulated maximum temperature rise and magnitude of flow velocity with respect to the chamber height for on-resonant conditions with 50 mW laser power. Both *x* axes are in log scale.

S8: Movies

Supplementary video V1

Flow velocity comparison for three different input wavelengths.

With the input laser power fluctuating around 405 mW, the flow velocity increased significantly when approaching the quasi-BIC resonance (1544.3 nm), as indicated by the tracer particles (500 nm polystyrene beads). The videos in all three panels were accelerated by 2.4 times (24 FPS).

Supplementary video V2

Generation of the ring-shaped distribution for the on-resonant condition at high power with the CTAC concentration of 5 mM.

At 360 mW, a small ring shape already appeared. When gradually increasing the input laser power to 420 mW, the increased positive thermophoresis overcame the attractive depletion-attraction

force, and particles were rapidly repelled out to generate the large ring-shaped distribution. The video was accelerated by 3 times (30 FPS).

Supplementary video V3

Rapid particle transport and aggregation for 200 nm polystyrene beads.

The input wavelength is about 1 nm away from resonance and the laser power is at 432 mW. Due to the small size of the tracer particles (i.e., weak fluorescence), the exposure time for imaging is increased to 200 ms and the video was accelerated by 3 times (15 FPS).

References

1. Vaveliuk, P., Ruiz, B. & Lencina, A. Limits of the paraxial approximation in laser beams. *Optics Letters* **32**, 927–929 (2007).
2. Ndukaife, J. C., *et al.* A. Long-range and rapid transport of individual nano-objects by a hybrid electrothermoplasmonic nanotweezer. *Nature Nanotechnology* **11**, 53-59 (2016).
3. Crocker, J. C. & Grier, D. G. Methods of Digital Video Microscopy for Colloidal Studies. *Journal of Colloid and Interface Science* **179**, 298–310 (1996).
4. Allan, Daniel B., Caswell, Thomas, Keim, Nathan C., van der Wel, Casper M., & Verweij, Ruben W. (2021). soft-matter/trackpy: Trackpy v0.5.0 (v0.5.0). Zenodo. https://doi.org/10.5281/zenodo.4682814
5. Yang, S., *et al*. Nanoparticle trapping in a quasi-BIC system. *ACS Photonics* **8**, 1961-1971 (2021).
6. Braun, D., & Libchaber, A. Trapping of DNA by thermophoretic depletion and convection. *Physical Review Letters* **89**, 188103 (2002).
7. Shoji, T., *et al*. Reversible photoinduced formation and manipulation of a two-dimensional closely packed assembly of polystyrene nanospheres on a metallic nanostructure. *The Journal of Physical Chemistry C* **117**, 2500-2506 (2013).
